# Association between weekend catch-up outdoor duration and prevalence of myopia: evidence from a cross-sectional, multi-center study in China

**DOI:** 10.1186/s12889-024-20466-0

**Published:** 2024-10-25

**Authors:** Lu Ye, Ying Wang, Ying Sun, Wu-jun Li, Guo-yun Zhang, Wen-jun Wang, Mei-xia Ren, Jun-cheng Gao, Guan-chen Liu, Yi-ming Guo, Juan Huang, Xin-xin Lu, Jie Min, Tuan-zheng Ran, Si-xuan Li, Zi-tong He, Qi-ya Jing, Pei-quan Wang, Liu-qing Qu, Yan-qi Yang, Pan Ge, Jian Zhang, Mo-qi Lv, Dang-xia Zhou

**Affiliations:** 1 Shaanxi Eye Hospital, Xi’an People’s Hospital (Xi’an Fourth Hospital), Xi’an, 710004 China; 2 Health Science Center, Xi’an Jiaotong University, Xi’an, 710061 China; 3 Department of Gynecology and Obstetrics, The Second Affiliated Hospital of Xi’an Jiaotong University, Xi’an, 710049 China; 4 Department of Pathology, Medical School, Xi’an Jiaotong University, 76 Yanta West Road, Xi’an, 710061 China; 5 Key Laboratory of Environment and Genes Related to Diseases, Ministry of Education, Xi’an, 710061 China; 6 Traditional Chinese Medicine Hospital of Yulin, Yulin Eye Hospital, Yulin, 719000 China; 7 Northwest A&F University, Xianyang, 712100 China; 8 School of Life Science and Technology, Xi’an Jiaotong University, Xi’an, 710049 China

**Keywords:** Weekend catch-up outdoor duration, Myopia, Cross-sectional study

## Abstract

**Background:**

This study aimed at investigating the relationship between the weekend catch-up outdoor duration (WCOD) and prevalence of myopia among students in China.

**Methods:**

This cross-sectional study recruited participants in 107 schools (six cities, 30 districts) from China from May to June 2021. Demographic characteristics (age, grade, sex, ethnicity, BMI, resident, and parents’ myopia), optically habits (bad writing habits, working/studying time per day, continuous working/studying time per day, and screen time per day) and outdoor duration (weekday and weekend) were obtained from questionnaire. WCOD was defined as outdoor time 1 h longer on weekends than on weekdays. Spherical equivalent (SE) of refractive error were measured with non-cycloplegic refraction. Adjusted multivariate logistic regression analysis was performed to evaluate the relationship between WCOD and prevalence of myopia.

**Results:**

Students with myopia had shorter WCOD compared with those without myopia (*P* < 0.001). Adjusted multivariate logistic regression analyses showed negative associations between WCOD and prevalence of myopia in Chinese students, especially in students with WCOD of 2–3 h (OR = 0.577, *P* < 0.001) and 3–4 h (OR = 0.571, *P* = 0.004) when the weekday outdoor duration was 0.5–1 h, as well as students with WCOD of 2–3 h (OR = 0.614, *P* = 0.003) when the weekday outdoor duration was 1–2 h. Similar results were observed in students with high myopia. Students with high myopia had shorter WCOD compared with those without high myopia (*P* = 0.001). Negative associations between WCOD and prevalence of high myopia were significant in students with WCOD of 1–2 h when the weekday outdoor duration was < 0.5 h (OR = 0.585, *P* = 0.007) and 0.5–1 h (OR = 0.537, *P* = 0.018).

**Conclusion:**

Our study, for the first time, reported that a WCOD have a potential to reduce the prevalence of myopia and high myopia in Chinese students.

**Supplementary Information:**

The online version contains supplementary material available at 10.1186/s12889-024-20466-0.

## Background

Myopia is the most common cause of visual impairment [1]. With the rapidly increasing prevalence over the past few decades [2–4], it is predicted that the overall number of people with myopia would reach 4758 million (49.8% of the world population) by 2050 [5]. Especially in children, 12.8 million children are diagnosed with visual impairment [6], and approximate half of these children live in China. In many East Asian countries including China, myopia has affected 80–90% students in school-leavers due to their special educational demands [7]. Therefore, the prevalence of myopia in Chinese school-aged students has alarmed for a global public health problem [8–10].

Prior evidence from cross-sectional studies, longitudinal cohort studies and systematic reviews indicated that outdoor time was a protective factor in reducing the incidence of myopia [11–13]. The protective effect might be result from various theories such as increased light exposure, increased depth of field, and release of dopamine from retina [14]. Nevertheless, most previous studies had only assessed the association between myopia and outdoor time [15–17]; the effect of different weekday and weekend outdoor duration on myopia in free-living individuals had not been studied. Additionally, considering the Chinese specific educational cultures including rigorous schooling system and the long hours spending in study in weekdays [18, 19], it’s hard for students to get enough outdoor time in weekdays. Hence, some students will use weekend catch-up outdoor duration (WCOD) to recover from outdoor loss incurred on weekdays. However, whether WCOD compensates for the accumulated outdoor time debt and modulates the effect of insufficient weekday outdoor time on prevalence of myopia need to be investigated.

Thus, we conducted a cross-sectional study of 6832 Chinese school-aged students to examine the association between WCOD and prevalence of myopia. In light of the acknowledged weekday outdoor time loss pervades school-aged students in China, our study was intended to provide evidences on which to base prevention and control of myopia for both public and professional health policies.

## Materials and methods

### Population

This was a cross-sectional analysis of data from Shaanxi province, north-western of China participants were school-aged children and adolescents recruited from 107 schools in six cities (30 districts) from May to June 2021. Individuals with grade < 4 were excluded in the present study, since their cognitive competence could not support them to finish the questionnaires. Additionally, we also excluded individuals: (1) who had retinopathy, prematurity, and Stickler or Marfan syndromes, (2) who rejected/failed to finish the ophthalmological examination. Participants and their guardians were emphasized the noncompulsory nature of participation and were informed to sign a written consent form. This study was approved by the Institutional Medical Ethics Committee of Xi’an Jiaotong University and followed the tenets of Declaration of Helsinki.

### Ophthalmological examination

Following the protocol reported in our previous study [20], ophthalmological examinations were performed using the non-cycloplegic auto-refractometry (auto-refractor KR-800; Topcon Co., Tokyo, Japan), and bilateral spherical equivalents (SE) of each participant were recorded. Myopia was defined as a SE of ≤-0.5 diopters (D) in the worse eye which had lower value of SE. High myopia was further divided as a SE of ≤-6.0D in the worse eye which had lower value of SE.

### Assessments of outdoor data and calculation of WCOD

Outdoor data were obtained from questionnaires (Supplementary file 1 & file 2): (1) “weekday outdoor duration = average hours of outdoor on weekdays in a week” and (2) “weekend outdoor duration = average hours of outdoor on weekends in a week”. The average outdoor duration was calculated using the following weighted mean value: (5 × weekday outdoor duration + 2 × weekend outdoor duration)/7 [21]. The WCOD was calculated as weekend outdoor duration minus weekday outdoor duration. Based on the previous study [22], we divided the weekend outdoor duration change into 3 group: time difference ≤ 1 h (treated as reference), less outdoor duration on weekends (> 1 h), and WCOD (> 1 h).

### Covariates

Based on clinical experience and available literature, questionnaire items (Supplementary file 1) addressed potential covariates including age (year), grade, sex (girls/boys), ethnicity (Han/non-Han), BMI (= Weight/Height^2^ (Kg/M^2^)), resident (village/urban), parents’ myopia (none of parents is myopia/at least one is myopia), number of bad writing habits (containing ①when reading or writing, the distance between the eyes and the table is less than 33 cm, ②when reading or writing, the distance of the chest from the table is less than the width of a punch, ③when writing, the distance between the hand and the tip of the pen is less than 3.3 cm, ④often tilt your head when reading or writing, ⑤often read or write on your stomach), working/studying time per day (≤ 6 h/6–8 h/8–10 h/>10 h), continuous working/studying time per day (≤ 1 h/1–2 h/2–3 h/>3 h), screen time per day (≤ 0.5 h/0.5–1 h/1–2 h/>2 h).

### Statistical analyses

The main objective of our study was to investigate the relationship of the prevalence of myopia with WCOD.

Kolmogorov-Smirnov test was used to assess the normality of the data. Normally distributed continuous variables were presented as mean ± standard deviation (SD) and were analyzed using Student’s t-test. Non-normally distributed continuous variables were presented as median (IQR) and were analyzed using Mann-Whitney U test. Categorical variables were presented as frequencies of the total and were analyzed using Chi-square test.

Univariate logistic regression analyses were performed to examine associations with shorter (> 1 h shorter than weekday outdoor)/longer (> 1 h longer than weekday outdoor) weekend outdoor duration vs. constant (within 1 h) weekend outdoor duration. Thereafter, after adjusting for covariates containing age, grade, sex, ethnicity, BMI, resident, parents’ myopia, number of bad writing habits, working/studying time per day, continuous working/studying time per day, and screen time per day, multivariate logistic regression analyses were conducted to assess associations between WCOD and the prevalence of myopia. Odds ratio (OR) and 95% confidence interval (CI) were presented in logistic regression analyses. Further stratified analysis investigated associations between WCOD and the prevalence of high myopia.

All statistical analyses were performed using SPSS (V.18.0, SPSS Inc, IL, USA). A *P* < 0.05 was considered to be statistically significant.

## Results

### Demographic characteristics of the study population

9424 individuals were recruited in this study. 2592 individuals were excluded, 2096 of which with grade < 4, 115 of which with retinopathy, prematurity, Strickler or Marfan syndromes, 14 of which failed to finish the ophthalmological examination due to the ocular trauma, 27 of which rejected to perform the ophthalmological examination, 53 of which were absent on the examination day, 287 of which with missing data > 30%. Finally, 6832 students were included in the present study with a median (IQR) age of 11.2 (8.4, 13.7) years and a median (IQR) grade of 9 (6, 12). Boys accounted for 51.2% (3496/6832) and Han ethnicity accounted for the majority (6764/6832, 99.0%).

### Ophthalmological condition

The overall prevalence of myopia was 70.7% (4832/6832). Individuals with myopia had shorter weekday outdoor duration (1.143 ± 0.828 h vs. 1.236 ± 0.851 h, *P* < 0.001), shorter weekend outdoor duration (1.500(1.500, 2.500) hours vs. 2.500(1.500, 2.500) hours, *P* < 0.001), shorter average outdoor duration (1.214(0.893, 1.786) hours vs. 1.250(0.964, 1.821) hours, *P* < 0.001), and shorter WCOD (0.000(-1.000, 1.000) hour vs. 0.000(0.000, 1.000) hour, *P* < 0.001) than those without myopia (Table 1).

**Table 1:** Outdoor data of participants with and without myopia (*N* = 6832)

	median (IQR)^#2^		*P* value	mean ± SD^#3^		*P* value
	Non-myopia (*N* = 2000)	Myopia (*N* = 4832)		Non-myopia (*N* = 2000)	Myopia (*N* = 4832)	
**Weekday outdoor duration**,** hour**^**#1**^	0.750(0.750, 1.500)	0.750(0.750, 1.500)	< 0.001*	1.236 ± 0.851	1.143 ± 0.828	< 0.001*
**Weekend outdoor duration**,** hour**	2.500(1.500, 2.500)	1.500(1.500, 2.500)	< 0.001*	2.222 ± 1.194	1.855 ± 1.161	< 0.001*
**Average outdoor duration**,** hour**	1.250(0.964, 1.821)	1.214(0.893, 1.786)	< 0.001*	1.518 ± 0.801	1.347 ± 0.783	< 0.001*
**Weekend catch-up outdoor duration**,** hour**	0.000(0.000, 1.000)	0.000(-1.000, 1.000)	< 0.001*	0.188 ± 1.244	-0.054 ± 1.202	< 0.001*

#1: This was non-normally distributed continuous variable. However, the median (IQR) of myopia group were same to that of non-myopia group, hence the calculation of mean ± SD was added

#2: Data was analyzed using non-parametric test

#3: Data was analyzed using Student’s t-test

*: *P* < 0.05

Moreover, as shown in Table 2, individuals with myopia, compared with whose with non-myopia, had older median (IQR) age (12.4 [9.9, 14.4] vs. 8.3 [7.1, 10.4]; *P* < 0.001), higher median (IQR) grade (10 [8, 13] vs. 7 [5, 10]; *P* < 0.001), higher median (IQR) BMI (18.667 [16.442, 21.083] vs. 17.361 [15.278, 20.196]; *P* < 0.001), higher incidence of girls (50.5% vs. 44.8%; *P* < 0.001), higher incidence of parents’ myopia (47.8% vs. 42.5%; *P* < 0.001), higher incidence of bad writing habits (68.3% vs. 60.7%; *P* < 0.001), higher incidence of longer (> 8 h) working/studying time per day (37.5% vs. 14.1%; *P* < 0.001), higher incidence of longer (> 2 h) continuous working/studying time per day (47.2% vs. 31.8%; *P* < 0.001), and higher incidence of longer (> 1 h) screen time per day (39.4% vs. 25.5%; *P* < 0.001).

**Table 2:** Characteristics of participants with and without myopia (*N* = 6832)

	Non-myopia (*N* = 2000)	Myopia (*N* = 4832)	*P* value
**Grade**,** median (IQR)**^**#1**^	7 (5, 10)	10 (8, 13)	< 0.001*
**Age**,** median (IQR)**,** year**^**#1**^	8.3 (7.1, 10.4)	12.4 (9.9, 14.4)	< 0.001*
**Ethnicity**,** No. (%)**^**#2**^			
Non-Han	24 (1.2)	44 (0.9)	0.352
Han	1976 (98.8)	4788 (99.1)	
**Sex**,** No. (%)**^**#2**^			
Boys	1104 (55.2)	2392 (49.5)	< 0.001*
Girls	896 (44.8)	2440 (50.5)	
**BMI**,** median (IQR)**^**#1**^	17.361 (15.278, 20.196)	18.667 (16.442, 21.083)	< 0.001*
**Resident**,** No. (%)**^**#2**^			
Village	874 (43.7)	2145 (44.4)	0.600
Urban	1126 (56.3)	2687 (55.6)	
**Parents’ myopia**,** No. (%)**^**#2**^			
None of them is myopia	1149 (57.5)	2524 (52.2)	< 0.001*
At least one is myopia	851 (42.5)	2308 (47.8)	
**Have any bad writing habits** ^**#3**^ **? No. (%)** ^**#2**^			
Have little bad writing habit	787 (39.3)	1533 (31.7)	< 0.001*
Have a kind of bad writing habit	415 (20.8)	991 (20.5)	
Have two kinds of bad writing habits	322 (16.1)	865 (17.9)	
Have three kinds of bad writing habits	308 (15.4)	841 (17.4)	
Have four kinds of bad writing habits	114 (5.7)	386 (8.0)	
Have five kinds of bad writing habits	54 (2.7)	216 (4.5)	
**Working/Studying time per day**,** No. (%)**^**#2**^			
≤ 6 h	526 (26.3)	529 (10.9)	< 0.001*
6–8 h	1192 (59.6)	2492 (51.6)	
8–10 h	196 (9.8)	1132 (23.4)	
> 10 h	86 (4.3)	679 (14.1)	
**Continuous working/studying time per day**,** No. (%)**^**#2**^			
≤ 1 h	496 (24.8)	643 (13.3)	< 0.001*
1–2 h	868 (43.4)	1909 (39.5)	
2–3 h	439 (21.9)	1370 (28.4)	
> 3 h	197 (9.9)	910 (18.8)	
**Screen time per day**,** No. (%)**^**#2**^			
≤ 0.5 h	641 (32.1)	1092 (22.6)	< 0.001*
0.5–1 h	849 (42.4)	1834 (38.0)	
1–2 h	376 (18.8)	1184 (24.5)	
> 2 h	134 (6.7)	722 (14.9)	

#1: Data was analyzed using non-parametric test

#2: Data was analyzed using Chi-square test

#3: Bad writing habits include ①when reading or writing, the distance between the eyes and the table is less than 33 cm, ②when reading or writing, the distance of the chest from the table is less than the width of a punch, ③when writing, the distance between the hand and the tip of the pen is less than 3.3 cm, ④often tilt your head when reading or writing, ⑤often read or write on your stomach

*: *P* < 0.05

### Weekend outdoor duration changes and myopia

**Fig. 1:**
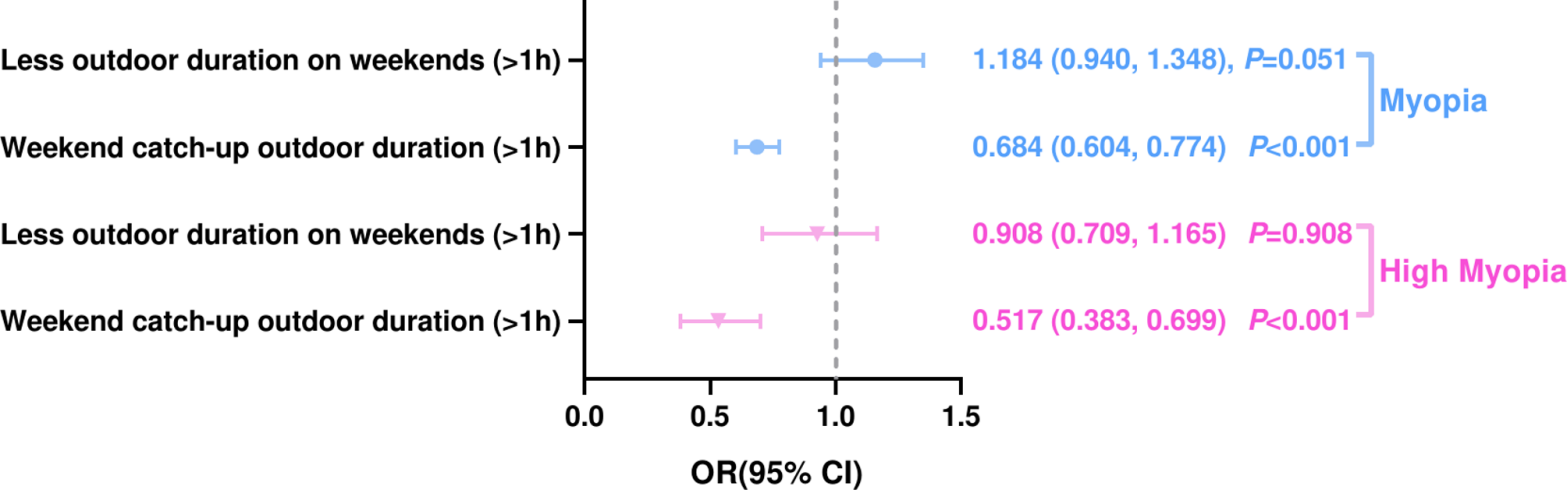
Univariate analysis for assessing the association of weekend outdoor duration change with myopia and high myopia

Univariate analysis was conducted to assess associations of weekend vs. weekday outdoor duration and prevalence of myopia. Lower myopia prevalence (OR = 0.684, 95%CI: 0.604–0.774, *P* < 0.001) were significantly associated with longer (> 1 h) WCOD (Fig. 1). However, no association (OR = 1.184, 95%CI: 0.940–1.348, *P* = 0.051) was found between myopia prevalence and less outdoor duration on weekends (> 1 h).

### Association of WCOD with myopia

After excluded participants whose outdoor more than 1 h longer on weekdays than weekends, multivariate logistic regression analyses were performed to examine the associations of WCOD with prevalence of myopia.

In the complete sample, longer WCOD was significantly associated with lower prevalence of myopia (OR = 0.766, 95%CI: 0.672–0.873, *P* < 0.001, Table 3). Additionally, subgroups were created based on weekday outdoor duration, and similar associations between longer WCOD and lower prevalence of myopia were seen in individuals with ≤ 0.5 h of weekday outdoor duration (OR = 0.678, 95%CI: 0.497–0.925, *P* = 0.014, Table 3), individuals with 0.5–1 h of weekday outdoor duration (OR = 0.695, 95%CI: 0.577–0.836, *P* < 0.001, Table 3), and individuals with 1–2 h of weekday outdoor duration (OR = 0.753, 95%CI: 0.573–0.991, *P* = 0.043, Table 3). This tendency seemed to be time dependent.

**Table 3:** Adjusted multivariate logistic regression analysis^#^ for assessing the association between weekend catch-up outdoor time and myopia (*N* = 5467)

Weekend catch-up outdoor time(> 1 h)	Myopia (OR (95% CI))	*P* value
**Complete sample**		
No (*n* = 3288)	Reference	-
Yes (*n* = 2179)	0.766 (0.672, 0.873)	< 0.001*
**Weekday outdoor time ≤ 0.5 h**		
No (*n* = 661)	Reference	-
Yes (*n* = 529)	0.678 (0.497, 0.925)	0.014*
**0.5 h < Weekday outdoor time ≤ 1 h**		
No (*n* = 1635)	Reference	-
Yes (*n* = 1076)	0.695 (0.577, 0.836)	< 0.001*
**1 h < Weekday outdoor time ≤ 2 h**		
No (*n* = 678)	Reference	-
Yes (*n* = 429)	0.753 (0.573, 0.991)	0.043*
**2 h < Weekday outdoor time ≤ 3 h**		
No (*n* = 103)	Reference	-
Yes (*n* = 84)	1.713 (0.876, 3.352)	0.116
**Weekday outdoor time > 3 h**		
No (*n* = 211)	Reference	-
Yes (*n* = 61)	1.585 (0.971, 2.441)	0.088

#: Data was analyzed using adjusted multivariate logistic regression analysis and covariates used in the adjusted model contained age, grade, sex, ethnicity, BMI, resident, parents’ myopia, number of bad writing habits, working/studying time per day, continuous working/studying time per day, and screen time per day

*: *P* < 0.05

Participants with WCOD were further divided into those who had 1–2 h, 2–3 h, 3–4 h, and > 4 h of WCOD. Adjusted multivariate logistic regression analysis showed that lower prevalence of myopia was significantly associated with WCOD of 2–3 h (OR = 0.761, 95%CI: 0.646–0.896, *P* = 0.001, Table 4), 3–4 h (OR = 0.652, 95%CI: 0.519–0.818, *P* < 0.001, Table 4), and > 4 h (OR = 0.626, 95%CI: 0.451–0.870, *P* = 0.005, Table 4) in the complete sample.

**Table 4:** Adjusted multivariate logistic regression analysis^#^ for assessing the association between the duration of weekend catch-up outdoor and myopia (*N* = 5467)

Weekend catch-up outdoor time	Myopia (OR (95% CI))	*P* value
**Complete sample**		
Normal (*n* = 3288)	Reference	
1 h < time ≤ 2 h (*n* = 1443)	0.947 (0.825, 1.086)	0.434
2 h < time ≤ 3 h (*n* = 497)	0.761 (0.646, 0.896)	0.001*
3 h < time ≤ 4 h (*n* = 192)	0.652 (0.519, 0.818)	< 0.001*
time > 4 h (*n* = 47)	0.626 (0.451, 0.870)	0.005*
**Weekday outdoor time ≤ 0.5 h**		
Normal (*n* = 661)	Reference	
1 h < time ≤ 2 h (*n* = 330)	1.261 (0.603, 2.636)	0.538
2 h < time ≤ 3 h (*n* = 117)	0.987 (0.463, 2.103)	0.972
3 h < time ≤ 4 h (*n* = 35)	0.556 (0.246, 1.259)	0.159
time > 4 h (*n* = 47)	0.933 (0.328, 2.650)	0.896
**0.5 h < Weekday outdoor time ≤ 1 h**		
Normal (*n* = 1635)	Reference	
1 h < time ≤ 2 h (*n* = 725)	0.795 (0.621, 1.016)	0.067
2 h < time ≤ 3 h (*n* = 194)	0.577 (0.439, 0.758)	< 0.001*
3 h < time ≤ 4 h (*n* = 157)	0.571 (0.389, 0.839)	0.004*
time > 4 h (*n* = 0)	-	-
**1 h < Weekday outdoor time ≤ 2 h**		
Normal (*n* = 678)	Reference	
1 h < time ≤ 2 h (*n* = 243)	0.829 (0.653, 1.052)	0.124
2 h < time ≤ 3 h (*n* = 186)	0.614 (0.446, 0.844)	0.003*
time > 3 h (*n* = 0)	-	-
**2 h < Weekday outdoor time ≤ 3 h**		
Normal (*n* = 103)	Reference	
1 h < time ≤ 2 h (*n* = 84)	1.713 (0.876, 3.352)	0.116
time > 2 h (*n* = 0)	-	-
**Weekday outdoor time > 3 h**		
Normal (*n* = 211)	Reference	
1 h < time ≤ 2 h (*n* = 61)	1.585 (0.971, 2.441)	0.088
time > 2 h (*n* = 0)	-	-

#: Data was analyzed using adjusted multivariate logistic regression analysis and covariates used in the adjusted model contained age, grade, sex, ethnicity, BMI, resident, parents’ myopia, number of bad writing habits, working/studying time per day, continuous working/studying time per day, and screen time per day

*: *P* < 0.05

In subgroups, similar associations of lower prevalence of myopia were also seen with individuals with WCOD of 2–3 h (OR = 0.577, 95%CI: 0.439–0.758, *P* < 0.001, Table 4) and 3–4 h (OR = 0.571, 95%CI: 0.389–0.839, *P* = 0.004, Table 4) when the weekday outdoor duration was 0.5–1 h, as well as individuals with WCOD of 2–3 h (OR = 0.614, 95%CI: 0.446–0.844, *P* = 0.003, Table 4) when the weekday outdoor duration was 1–2 h.

### Stratified analyses

The prevalence of high myopia in the present study was 4.9% (337/6832). Individuals with high myopia had shorter weekday outdoor duration (1.039 ± 0.785 h vs. 1.177 ± 0.838 h, *P* = 0.003), shorter weekend outdoor duration (1.500(0.500, 1.500) hours vs. 1.500(1.500, 2.500) hours, *P* < 0.001), shorter average outdoor duration (0.964(0.679, 1.500) hours vs. 1.250(0.964, 1.786) hours, *P* < 0.001), and shorter WCOD (0.000(-1.000, 0.000) hour vs. 0.000(-1.000, 1.000) hour, *P* = 0.001) than those without high myopia (Table 5).

**Table 5:** Outdoor data and of participants with and without high myopia (*N* = 6832)

	median (IQR)^#2^		*P* value	mean ± SD^#3^		*P* value
	Non high myopia^#4^ (*N* = 6495)	High Myopia (*N* = 337)		Non high myopia^#4^ (*N* = 6495)	High Myopia (*N* = 337)	
**Weekday outdoor duration**,** hour**^**#1**^	0.750(0.750, 1.500)	0.750(0.750, 1.500)	0.001*	1.177 ± 0.838	1.039 ± 0.785	0.003*
**Weekend outdoor duration**,** hour**	1.500(1.500, 2.500)	1.500(0.500, 1.500)	< 0.001*	1.983 ± 1.185	1.565 ± 1.064	< 0.001*
**Average outdoor duration**,** hour**	1.250(0.964, 1.786)	0.964(0.679, 1.500)	< 0.001*	1.407 ± 0.794	1.190 ± 0.730	< 0.001*
**Weekend catch-up outdoor duration**,** hour**	0.000(-1.000, 1.000)	0.000(-1.000, 0.000)	0.001*	0.028 ± 1.222	-0.199 ± 1.157	0.001*

#1: This was non-normally distributed continuous variable. However, the median (IQR) of myopia group were same to that of non high myopia group, hence the calculation of mean ± SD was added

#2: Data was analyzed using non-parametric test

#3: Data was analyzed using Student’s t-test

#4: Participants included low/moderate myopia as well as non-myopia

*: *P* < 0.05

Additionally, as shown in Table 6, individuals with high myopia, compared with whose with non high myopia (including low/moderate myopia as well as non-myopia), had older median (IQR) age (14.8 [13.0, 16.5] vs. 10.9 [8.3, 13.5]; *P* < 0.001), higher median (IQR) grade (12 [9, 15] vs. 9 [6, 12]; *P* < 0.001), higher median (IQR) BMI (19.234 [16.937, 21.565] vs. 17.959 [15.816, 20.703]; *P* < 0.001), higher incidence of girls (56.4% vs. 48.4%; *P* = 0.004), higher incidence of parents’ myopia (5.3% vs. 3.2%; *P* = 0.037), higher incidence of bad writing habits (69.7% vs. 65.9%; *P* < 0.001), higher incidence of longer (> 8 h) working/studying time per day (57.9% vs. 29.3%; *P* < 0.001), higher incidence of longer (> 2 h) continuous working/studying time per day (56.7% vs. 42.0%; *P* < 0.001), and higher incidence of longer (> 1 h) screen time per day (51.9% vs. 34.5%; *P* < 0.001).

**Table 6:** Characteristics of participants with and without high myopia (*N* = 6832)

	Non high myopia^#4^ (*N* = 6495)	High myopia (*N* = 337)	*P* value
**Grade**,** median (IQR)**^**#1**^	9 (6, 12)	12 (9, 15)	< 0.001*
**Age**,** median (IQR)**,** year**^**#1**^	10.9 (8.3, 13.5)	14.8 (13.0, 16.5)	< 0.001*
**Ethnicity**,** No. (%)**^**#2**^			
Non-Han	24 (0.4)	4 (1.2)	0.785
Han	6471 (99.6)	333 (98.8)	
**Sex**,** No. (%)**^**#2**^			
Boys	3349 (51.6)	147 (43.6)	0.004*
Girls	3146 (48.4)	190 (56.4)	
**BMI**,** median (IQR)**^**#1**^	17.959 (15.816, 20.703)	19.234 (16.937, 21.565)	< 0.001*
**Resident**,** No. (%)**^**#2**^			
Village	2863 (44.1)	156 (46.3)	0.426
Urban	3632 (55.9)	181 (53.7)	
**Parents’ high myopia**,** No. (%)**^**#2**^			
None of them are high myopia	6284 (96.8)	319 (94.7)	0.037*
At least one is high myopia	211 (3.2)	18 (5.3)	
**Have any bad writing habits** ^**#3**^ **? No. (%)** ^**#2**^			
Have little bad writing habit	2218 (34.1)	102 (30.3)	< 0.001*
Have a kind of bad writing habit	1337 (20.6)	69 (20.5)	
Have two kinds of bad writing habits	1116 (17.2)	71 (21.1)	
Have three kinds of bad writing habits	1096 (16.9)	53 (15.7)	
Have four kinds of bad writing habits	472 (7.3)	28 (8.3)	
Have five kinds of bad writing habits	256 (3.9)	14 (4.1)	
**Working/Studying time per day**,** No. (%)**^**#2**^			
≤ 6 h	1043 (16.0)	12 (3.5)	< 0.001*
6–8 h	3554 (54.7)	130 (38.6)	
8–10 h	1225 (18.9)	103 (30.6)	
> 10 h	673 (10.4)	92 (27.3)	
**Continuous working/studying time per day**,** No. (%)**^**#2**^			
≤ 1 h	1116 (17.2)	23 (6.8)	< 0.001*
1–2 h	2654 (40.8)	123 (36.5)	
2–3 h	1713 (26.4)	96 (28.5)	
> 3 h	1012 (15.6)	95 (28.2)	
**Screen time per day**,** No. (%)**^**#2**^			
≤ 0.5 h	1689 (26.0)	44 (13.1)	< 0.001*
0.5–1 h	2565 (39.5)	118 (35.0)	
1–2 h	1445 (22.2)	115 (34.1)	
> 2 h	796 (12.3)	60 (17.8)	

#1: Data was analyzed using non-parametric test

#2: Data was analyzed using Chi-square test

#3: Bad writing habits include ①when reading or writing, the distance between the eyes and the table is less than 33 cm, ②when reading or writing, the distance of the chest from the table is less than the width of a punch, ③when writing, the distance between the hand and the tip of the pen is less than 3.3 cm, ④often tilt your head when reading or writing, ⑤often read or write on your stoma

#4: Participants included low/moderate myopia as well as non-myopia

*: *P* < 0.05

Univariate analysis indicated that lower prevalence of high myopia (OR = 0.517, 95%CI: 0.383–0.699, *P* < 0.001) were significantly associated with longer (> 1 h) WCOD (Fig. 1). No association (OR = 0.908, 95%CI: 0.709–1.165, *P* = 0.908) was seen between high myopia prevalence and less outdoor duration on weekends (> 1 h).

Adjusted multivariate logistic regression analyses showed that, longer outdoor duration on weekend (> 1 h) was significantly associated with lower prevalence of high myopia (OR = 0.603, 95%CI: 0.444–0.818, *P* = 0.001, Table 7) in the complete sample. Moreover, these associations were only significant in subgroup with ≤ 0.5 h (OR = 0.507, 95%CI: 0.383–0.747, *P* = 0.004, Table 7) and 0.5–1 h (OR = 0.478, 95%CI: 0.301–0.757, *P* = 0.002, Table 7) of weekday outdoor duration.

**Table 7:** Adjusted multivariate logistic regression analysis^#^ for assessing the association between weekend catch-up outdoor time and high myopia (*N* = 5467)

Weekend catch-up outdoor time(> 1 h)	High myopia (OR (95% CI))	*P* value
**Complete sample**		
No (*n* = 3288)	Reference	-
Yes (*n* = 2179)	0.603 (0.444, 0.818)	0.001*
**Weekday outdoor time ≤ 0.5 h**		
No (*n* = 661)	Reference	-
Yes (*n* = 529)	0.507 (0.383, 0.747)	0.004*
**0.5 h < Weekday outdoor time ≤ 1 h**		
No (*n* = 1635)	Reference	-
Yes (*n* = 1076)	0.478 (0.301, 0.757)	0.002*
**1 h < Weekday outdoor time ≤ 2 h**		
No (*n* = 678)	Reference	-
Yes (*n* = 429)	0.753 (0.573, 1.091)	0.053
**2 h < Weekday outdoor time ≤ 3 h**		
No (*n* = 103)	Reference	-
Yes (*n* = 84)	0.711 (0.229, 2.202)	0.554
**Weekday outdoor time > 3 h**		
No (*n* = 211)	Reference	-
Yes (*n* = 61)	0.790 (0.177, 4.521)	0.997

#: Data was analyzed using adjusted multivariate logistic regression analysis and covariates used in the adjusted model contained age, grade, sex, ethnicity, BMI, resident, parents’ high myopia, number of bad writing habits, working/studying time per day, continuous working/studying time per day, and screen time per day

*: *P* < 0.05

Further multivariate logistic regression analyses were conducted to identify the association between WCOD and high myopia. Lower prevalence of high myopia was significantly associated with WCOD of 1–2 h (OR = 0.693, 95%CI: 0.493–0.975, *P* = 0.035, Table 8) and 2–3 h (OR = 0.213, 95%CI: 0.086–0.523, *P* = 0.001, Table 8) in the complete sample.

**Table 8:** Adjusted multivariate logistic regression analysis^#^ for assessing the association between the duration of weekend catch-up outdoor and high myopia (*N* = 5467)

Weekend catch-up outdoor time	High myopia (OR (95% CI))	*P* value
**Complete sample**		
Normal (*n* = 3288)	Reference	
1 h < time ≤ 2 h (*n* = 1443)	0.693 (0.493, 0.975)	0.035*
2 h < time ≤ 3 h (*n* = 497)	0.213 (0.086, 0.523)	0.001*
3 h < time ≤ 4 h (*n* = 192)	0.654 (0.283, 1.514)	0.321
time > 4 h (*n* = 47)	2.170 (0.745, 6.320)	0.155
**Weekday outdoor time ≤ 0.5 h**		
Normal (*n* = 661)	Reference	
1 h < time ≤ 2 h (*n* = 330)	0.585 (0.363, 0.824)	0.007*
2 h < time ≤ 3 h (*n* = 117)	0.252 (0.060, 1.069)	0.062
3 h < time ≤ 4 h (*n* = 35)	0.515 (0.004, 1.077)	0.998
time > 4 h (*n* = 47)	1.690 (0.549, 5.200)	0.360
**0.5 h < Weekday outdoor time ≤ 1 h**		
Normal (*n* = 1635)	Reference	
1 h < time ≤ 2 h (*n* = 725)	0.537 (0.322, 0.898)	0.018*
2 h < time ≤ 3 h (*n* = 194)	0.697 (0.295, 1.647)	0.411
3 h < time ≤ 4 h (*n* = 157)	0.848 (0.564, 1.273)	0.426
time > 4 h (*n* = 0)	-	-
**1 h < Weekday outdoor time ≤ 2 h**		
Normal (*n* = 678)	Reference	
1 h < time ≤ 2 h (*n* = 243)	0.751 (0.297, 1.901)	0.546
2 h < time ≤ 3 h (*n* = 186)	0.489 (0.143, 1.669)	0.253
time > 3 h (*n* = 0)	-	-
**2 h < Weekday outdoor time ≤ 3 h**		
Normal (*n* = 103)	Reference	
1 h < time ≤ 2 h (*n* = 84)	0.711 (0.229, 2.202)	0.554
time > 2 h (*n* = 0)	-	-
**Weekday outdoor time > 3 h**		
Normal (*n* = 211)	Reference	
1 h < time ≤ 2 h (*n* = 61)	0.790 (0.177, 4.521)	0.997
time > 2 h (*n* = 0)	-	-

#: Data was analyzed using adjusted multivariate logistic regression analysis and covariates used in the adjusted model contained age, grade, sex, ethnicity, BMI, resident, parents’ high myopia, number of bad writing habits, working/studying time per day, continuous working/studying time per day, and screen time per day.

*: *P* < 0.05.

In subgroups, similar associations were only seen in individuals with WCOD of 1–2 h when the weekday outdoor duration was < 0.5 h (OR = 0.585, 95%CI: 0.363–0.824, *P* = 0.007, Table 8) and 0.5–1 h (OR = 0.537, 95%CI: 0.322–0.898, *P* = 0.018, Table 8).

## Discussion

This school-based cross-sectional study, for the first time, indicated that a longer WCOD in Chinese students was associated with a low prevalence of myopia and high myopia. Specifically, a WCOD of > 2 h/day was associated with reduced prevalence of myopia among students with short weekday outdoor duration (0.5–2 h/day). Moreover, similar and more narrow associations of WCOD were seen with high myopia. Students with a WCOD of 1–2 h/day showed associations with reduced prevalence of myopia among students with short weekday outdoor duration (≤ 1 h/day).

Outdoor activity is the main protective factor against myopia [23, 24]. In 2017, a meta-analysis summarized 25 articles, covering clinical trials, cohort studies and cross-sectional studies, and indicated that increased time outdoors is effective in reducing incidence of myopia [11]. Thereafter, another intervention experiment demonstrated that the prevalence of myopia was reduced from 49.4 to 46.1% in three years, when an outdoor time intervention program was implemented [25]. Thereafter, findings in the present study and some other cluster-randomized trials confirmed this effect [16, 26]. Further study found that more time spent in outdoor activity was associated with lower prevalence of myopia, but no similar effect was observed in indoor activity. This suggests that spending time outdoors, rather than physical activity, was protective [27].

Previous population-based studies and animal studies reported that the underlying mechanism of this protective effect might be that high light levels outdoor could affect the growth of axial eye, the release of dopamine, and Vitamin D synthesis [17]. The effect of light levels outdoor on myopia had been further supported by a study on seasonal variation and myopia, which found that myopia progression was slower in the summer [28]. Meanwhile, previous studies indicated that shorter light wavelengths (blue light) may be protective against myopia [17]. The prevent effect of active outside on myopia might attribute to receiving more daylight which is primarily composed of blue light.

Although the outdoor active has been confirmed to be a protective factor in myopia, students in China have insufficient outdoor time. The China 2018 Report Card on physical activity for children and youth demonstrated that only 13.1% students reported being physically active at 60 min daily [29]. This may due to the academic burden. In order to cope with the academic burden, Chinese students generally spend most of their time on study during weekdays [30]. Even on weekend with more free time, students will habitually choose screen time indoor over outdoor activity to make up for the lack of rest during weekdays, which may increase the prevalence of myopia [31]. Hence, it’s necessary to verify the effectiveness of WCOD in preventing myopia and strengthen the promotion of outdoor time in weekend.

Our study was the first to report the significant effect of WCOD on preventing myopia in Chinese students. In addition, considering that myopia is a multi-factor influenced disease, we incorporated a rich set of covariates to stablish our models, covering age, grade, sex, ethnicity, BMI, resident, parents’ myopia condition, bad writing habits, working/studying time, continuous working/studying time, and screen time, which were reported to affect prevalence of myopia and even high myopia [20, 32–38].

Despite the novel findings of our study from Chinese population, it has several limitations. First, cycloplegic autorefraction is considered the gold standard [39], and the refractive error was measured with non-cycloplegic autorefractor, which would overestimate the myopia prevalence of children [40]. However, it was the association of factors with myopia that was of interest instead of absolute values of myopic prevalence. Myopia and non-myopia shared the same measurement, indicating that the two groups had the same baseline, hence this might not pose serious measurement errors for the present study. Nevertheless, further study with cycloplegic autorefractor is still necessary. In addition, further study with non-cycloplegic autorefractor as well as more conservative thresholds for myopia could also be expect [41]. Second, outdoor duration was self-reported by the students through the recollection, thereby inducing a non-negligible memory bias. Future studies, using wearable devices to measure objectively outdoor data, are worth the wait. Third, since this was a cross-sectional study which was unable to evaluate the causality of the reported relationship, longitudinal investigations are needed to assess the underpinnings of the relationship between WCOD and myopia. Fourth, the present study was conducted in single region (northwestern China) which may affect the generalizability of our results. Further multi-regional and/or nationally cross-sectional studies with larger sample size should be conducted.

## Conclusion

In conclusion, we found that there were obvious relationships between WCOD and prevalence of myopia. Students with weekday outdoor duration in 0.5–2 h/day will reduce the prevalence of myopia when they have a WCOD of > 2 h/day. Students with weekday outdoor duration ≤ 1 h/day will reduce the prevalence of high myopia when they have a WCOD of 1–2 h/day. Our findings provided an enriched insight into the relationship between outdoor time (especially weekend catch-up outdoor duration) and childhood myopia.

## Electronic supplementary material

Below is the link to the electronic supplementary material.

Supplementary Material 1

## Data Availability

The datasets used and/or analysed during the current study are available from the corresponding author on reasonable request.

## Abbreviations

ANOVA: One-way analysis of variance
D: Diopter
IQR: Interquartile range
OR: Odds ratio
SD: Standard deviation
SE: Spherical equivalent
WCOD: Weekend catch-up outdoor duration
95%CI: 95% confidence interval
