# Supplementary material for: Association between weekend catch-up outdoor duration and prevalence of myopia: evidence from a cross-sectional, multi-center study in China
Source: BMC Public Health. 2024 Oct 25;24:2966. doi: 10.1186/s12889-024-20466-0 (PMC11515117; doi:10.1186/s12889-024-20466-0)
Supplement: Supplementary file 1 — Supplementary Material 1 [file 12889_2024_20466_MOESM1_ESM.pdf]

Supplementary Content

File 1. Questionnaire used in the present study.

File 2. Validation of the questionnaire.

File 1. Health Questionnaire of Myopia Prevention and Control in Shaanxi Children and Adolescents

（Please ask your parents to help complete this questionnaire if necessary）

① Basic information

1. Area of your school： ___________City ________County________District

2. School name: _______________

3. Student's grade: ____________Grade ________________Class

4. Student's ethnicity:

□Han nationality □Mongol nationality □Uyghur nationality □Zhuang nationality □Other ________________________

5. Student's national identification number______________________________

6. Student's date of birth: _____________Year_____________Month

7. Student's sex？

□Boy □Girl

8. Student's resident？

□Village □Urban

9. Student's height？

_______Meter(M)

10. Student's weight？

_______Kilogram(Kg)

11. Whether your father is myopia？ What's his diopters (D)?

□No □ ≥-0.5D & <-3.0D □≥-3.0D & <-6.0D □≥-6.0D □Not clear

12. Whether your mother is myopia？ What's her diopters (D)?

□No □ ≥-0.5D & <-3.0D □≥-3.0D & <-6.0D □≥-6.0D □Not clear

② Student's ophthalmological condition

1. When reading or writing, how long is the distance between the eyes and the table.

□<33cm □≥33cm

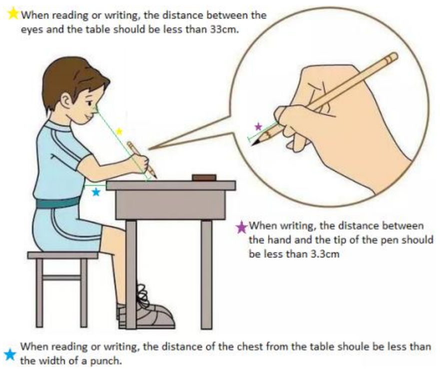

2. When reading or writing, how long is the distance of the chest from the table.

□<a width of a punch □≥a width of a punch

3. When writing, how long is the distance between the hand and the tip of the pen.

□<3.3cm □≥3.3cm

4. Do you often tilt your head when reading or writing？

□Never □Occasional □Constant □Always

5. Do you often read or write on your stomach？

□Never □Occasional □Constant □Always

6. In the past year, what's your working/studying time per day？

□≤6hours □>6hours & ≤8hours □>8hours & ≤10hours □>10hours

7. In the past year, how long is your continuous working/studying time per day？

□≤1hour □>1hour & ≤2hours □>2hours & ≤3hours □>3hours

8. In the past year, how long is your screen time per day？

□≤0.5hour □>0.5hour & ≤1hour □>1hours & ≤2hours □>2hours

9. In the past year, how long is your average hours of outdoor on weekdays per week？

□≤0.5hour □>0.5hour & ≤1hour □>1hours & ≤2hours □>2hours & ≤3hours □>3hours

10. In the past year, how long is your average hours of outdoor on weekends per week？

□≤1hour □>1hour & ≤2hours □>2hours & ≤3hours □>3hours & ≤4hours □>4hours

File 2. Validation of the questionnaire

1. Parents' validation of the questionnaire.

We randomly selected 66 parents from primary schools and middle schools, which were not included in the study. These parents were asked to provide feedback on how well the questionnaire was understood and how easy it was to fill out. Then, the questionnaire was determined according to the opinions of the parents.

2. Assessment of memory bias in the questionnaire

We randomly selected a primary school and a middle school. And 20 students are randomly selected from each grade. All the participants (N=240) were asked to complete the questionnaire independently. Meanwhile, they were also requested to wear wearable devices to measure objectively outdoor data. The following analysis found that there was no statistic difference between data from questionnaire and from wearable devices among participants with grade≥4 (shown in the following Table S1). Hence, when performing the formal experiment, in order to reduce data bias, we excluded individuals with grade<4.

Table S1 Outdoor data of participants measured with questionnaire and with wearable devices (N=240).

	median (IQR)		*P* value	mean±SD		*P* value
	Questionnaire	Wearable devices		Questionnaire	Wearable devices	
**Weekday outdoor duration, hour** [#1]						
**Grade1**	1.000(0.750, 1.500)	1.000(1.000, 1.500)	0.032*	1.484±1.773	1.604±1.303	0.039*
**Grade2**	1.000(0.750, 1.500)	1.000(1.000, 1.500)	0.044*	1.490±1.492	1.511±1.234	0.045*
**Grade3**	1.000(0.750, 1.500)	1.000(0.750, 1.500)	0.046*	1.447±1.288	1.450±1.209	0.051
**Grade4**	1.000(0.750, 1.500)	1.000(0.750, 1.500)	0.071	1.386±1.111	1.399±0.923	0.072

**Grade5**	0.750(0.750, 1.500)	0.750(0.750, 1.500)	0.182	1.236±0.848	1.239±0.809	0.179
**Grade6**	0.750(0.750, 1.500)	0.750(0.750, 1.500)	0.330	1.150±0.800	1.161±0.706	0.392
**Grade7**	0.750(0.750, 1.500)	0.750(0.750, 1.500)	0.711	1.163±0.840	1.155±0.822	0.724
**Grade8**	0.750(0.750, 1.500)	0.750(0.750, 1.500)	0.797	1.149±0.865	1.141±0.821	0.792
**Grade9**	0.750(0.750, 1.500)	0.750(0.750, 1.500)	0.783	1.155±0.788	1.149±0.724	0.779
**Grade10**	0.750(0.750, 1.500)	0.750(0.750, 1.500)	0.931	1.111±0.876	1.114±0.774	0.930
**Grade11**	0.750(0.750, 1.500)	0.750(0.750, 1.500)	0.916	1.107±0.811	1.113±0.759	0.909
**Grade12**	0.750(0.750, 1.500)	0.750(0.750, 1.500)	0.919	1.102±0.837	1.095±0.755	0.921
**Weekend outdoor duration, hour**						
**Grade1**	3.000(1.500, 3.500)	2.500(2.000, 3.500)	0.003*	2.472±1.427	2.597±1.338	0.006*
**Grade2**	2.500(1.500, 3.500)	2.500(2.000, 3.500)	0.009*	2.460±1.399	2.591±1.303	0.013*
**Grade3**	2.500(1.500, 3.500)	2.500(1.500, 3.500)	0.033*	2.499±1.396	2.704±1.300	0.035*
**Grade4**	2.500(1.500, 3.500)	2.500(1.500, 3.500)	0.059	2.366±1.200	2.390±1.289	0.072
**Grade5**	2.500(1.500, 3.500)	2.500(1.500, 3.500)	0.221	2.306±1.170	2.277±1.205	0.240
**Grade6**	1.500(1.500, 2.500)	1.500(1.500, 2.500)	0.339	2.152±1.159	2.137±1.144	0.313
**Grade7**	1.500(1.500, 2.500)	1.500(1.500, 2.500)	0.659	2.118±1.164	2.109±1.160	0.660
**Grade8**	1.500(1.500, 2.500)	1.500(1.500, 2.500)	0.492	2.175±1.211	2.181±1.201	0.521
**Grade9**	1.500(1.500, 2.500)	1.500(1.500, 2.500)	0.667	2.056±1.194	2.069±1.144	0.691
**Grade10**	1.500(1.500, 2.500)	1.500(1.500, 2.500)	0.932	1.997±1.216	2.002±1.209	0.903
**Grade11**	1.500(0.500, 2.500)	1.500(0.500, 2.500)	0.884	1.588±1.041	1.592±1.047	0.897
**Grade12**	1.500(0.500, 2.500)	1.500(0.500, 2.500)	0.889	1.567±1.073	1.561±1.069	0.890

#1: This was non-normally distributed continuous variable. However, the median of the questionnaire group were same to that of wearable devices group, hence the calculation of mean±SD was added.

*： *P*<0.05.
